# Molecular characterization of a bovine adenovirus type 7 (Bovine Atadenovirus F) strain isolated from a systemically infected calf in Germany

**DOI:** 10.1186/s12985-022-01817-y

**Published:** 2022-05-24

**Authors:** Sonja T. Jesse, Malgorzata Ciurkiewicz, Ute Siesenop, Ingo Spitzbarth, A. D. M. E. Osterhaus, Wolfgang Baumgärtner, Martin Ludlow

**Affiliations:** 1 Research Center Emerging Infections and Zoonoses (RIZ), University of Veterinary Medicine Hannover, D-30559 Hannover, Germany; 2 Department of Pathology, University of Veterinary Medicine Hanover, Hannover, Germany; 3 Department of Microbiology, University of Veterinary Medicine Hanover, Hannover, Germany; 4 Present Address: Faculty of Veterinary Medicine, Institute of Veterinary Pathology, Leipzig University, Leipzig, Germany

**Keywords:** Adenovirus, Bovine adenovirus type 7, Bovine Atadenovirus F, Calf, Fiber gene, Pathology

## Abstract

Bovine adenovirus 7 (BAdV-7) is an unclassified member of the genus Atadenovirus with a worldwide distribution and has been reported to induce clinical disease of varying severity in infected cattle, ranging from asymptomatic infections to severe enteric or respiratory disease. In this study, we used next-generation sequencing to obtain the first complete genome sequence of a European strain of BadV-7, from pooled spleen and liver tissue obtained from a deceased newborn Limousin calf. Histopathological analysis and electron microscopy showing systemic lesions in multiple organs with intranuclear amphophilic inclusions observed in endothelial cells in multiple peripheral tissues. Virus isolation was readily achieved from tissue homogenate using bovine esophagus cells (KOP-R), a strategy which should facilitate future in vitro or in vivo BAdV-7 studies. Phylogenetic analysis of available genome sequences of BAdV-7 showed that the newly identified strain groups most closely with a recent BAdV-7 strain, SD18-74, from the USA, confirming that this newly identified strain is a member of the Atadenovirus genus. The fiber gene was found to be highly conserved within BAdV-7 strains but was highly divergent in comparison to Ovine adenovirus 7 (OAdV-7) (39.56% aa sequence identity). Furthermore, we report a variable region of multiple tandem repeats between the coding regions of E4.1 and RH5 genes. In summary, the presented pathological and molecular characterization of this case suggests that further research into the worldwide molecular epidemiology and disease burden of BAdV-7 is warranted.

**Supplementary Information:**

The online version contains supplementary material available at 10.1186/s12985-022-01817-y.

## Introduction

The family Adenoviridae is comprised of six genera of non-enveloped double-stranded DNA viruses, which have been identified in a wide range of mammals, birds, reptiles, amphibians, and fish species [1]. Bovine adenoviruses are members of the genera Mastadenovirus (BAdV-1, -2, -3, and -10) and Atadenovirus (BAdV-4, -5, 6, -7 and -8) and have a worldwide distribution [2]. Bovine adenovirus 7 (BAdV-7) is a proposed member of the genus Atadenovirus [3, 4] and the ICTV have reported that BAdV-7 is related to members of the genus Atadenovirus, but has not yet approved a formal classification of this virus, presumably “Bovine Atadenovirus F”, as a new atadenovirus species, [1]. Formerly, the classification of bovine adenoviruses was based on serological reactivity and the designation was comprised of the natural host and a serial number (bovine adenovirus 1, 2 etc.) [3]. Variation in the serological cross-reactivity of bovine adenoviruses is based on specific antigenic determinants in the hexon proteins located in the virus capsid [3]. Following the restructuring of the Mastadenovirus and Atadenovirus genera according to molecular criteria such as genome organization and phylogenetic distances, several bovine adenoviruses have been renamed, grouped with others within one species or abolished. These changes are reflected in an updated species nomenclature, consisting of host taxon, virus genus and a letter (e.g., bovine mastadenovirus A) [1].

A recent study investigating the utility of using the presence of adenovirus in a sample as a potential marker for fecal contamination found that 13% of fecal, 100% of manure and 90% of urine samples from healthy cattle were positive for adenoviral DNA [5]. In addition, adenovirus DNA was frequently detected in soil, water runoff and fresh well water [5]. Adenovirus infections can remain asymptomatic in cattle, but have also been associated with a range of disease phenotypes, including infection of the respiratory tract [6, 7], alimentary tract [8–11] and systemic disease of newborn calves (weak calf syndrome) or yearling heifers [12–14]. BAdV-7 was initially isolated in bovine testicular (BT) cells in Japan in 1965 (Fukuroi strain) from a blood specimen obtained from a cattle affected by an acute febrile illness with diarrhea, rhinorrhea and conjunctivitis [15]. A further eight BAdV strains were isolated between 1966 and 1968 by the same group from blood, organ, feces and nasal secretions from cattle with similar clinical signs [15]. However, further reports on the worldwide epidemiology of BAdV-7 or associations with clinical disease are limited apart from two studies based on cases from the USA. BAdV-7 was isolated from a ten-month-old calf with fibrinopurulent pneumonia and from two newborn calves with pneumoenteritis [16] and in a separate study from eight healthy and sick calves, six of which were co-infected with bovine viral diarrhea virus (BVDV) [6]. Only a single report has been published on BAdV-7 infection in Europe in which serological studies showed that this is one of the most common viruses in cattle herds suffering from respiratory disease in Finland [17].

Although the association of BAdV-7 infection and clinical disease in cattle is unclear, a hexavalent bovine respiratory disease vaccine has been developed in Japan which contains a live attenuated and temperature sensitive vaccine strain of BadV-7 derived from the Fukuroi strain [18]. BAdV-7 naturally infects cattle, but also has been detected in sheep in Australia [9, 19] and is most closely antigenically related to ovine Adenovirus type 7 [6, 12, 19]. However, the complete genome sequences of the “Fukuroi” strain and the derivate live attenuated vaccine strain “TS-GT were only recently reported [18, 20]. This is augmented by a complete sequence for the SD18-74 strain which was isolated from pooled lung tissue from two 2-week-old calves from South Dakota, USA, that succumbed to acute enteric disease in 2018 [4]. In this study, we present the first molecular characterization of a complete genome sequence of a European BAdV-7 strain which was derived from a virus isolate obtained from a peracutely deceased calf in Germany. The BAdV-7 infection was systemic and was associated with necrotizing lesions in peripheral tissues. This analysis confirms the necessity to assign BAdV-7 to its own species within the Atadenovirus genus.

## Materials and methods

### Clinical presentation and pathology

A sudden onset of illness was observed in four newborn Limousin calves, which were housed in the same pen as their mothers on a farm in northern Germany. The animals ranged from one to sixteen days of age. The calves showed apathy, dehydration, and polydipsia and the three youngest animals died within twelve hours of onset of symptoms. The oldest calf had a protracted disease course with overt diarrhea but survived and recovered completely following symptomatic therapy. One of the younger calves (eight days old) died during transport to a veterinary clinic and was submitted for necropsy. Samples derived from various organs were collected, formalin fixed, stained with hematoxylin–eosin, and sectioned as previously described [21]. Samples for transmissible electron microscopy (TEM) were also processed by the pop off technique [22] and visualization was performed using an EM 10C transmission electron microscope (Carl Zeiss). Tissues were routinely tested for notifiable bacterial and viral pathogens (Additional file 1).

### Virus isolation and titration

Bovine esophagus cells (KOP-R) (kindly provided by Prof. Georg Herrler, Institute of Virology, University of Veterinary Medicine Hannover Foundation) were maintained in Dulbecco's Modified Eagle Medium (DMEM) (Gibco) supplemented with 10% fetal bovine serum and penicillin (100 IU/mL)/streptomycin (100 µg/mL). Liver tissue (200 mg) was homogenized with 600 µl of OptiMEM (Thermo Fisher Scientific), filtered through 0.45 µm spin filters and diluted in OptiMEM (1:10 (v/v) dilution). T25 tissue culture flasks of KOP-R (bovine esophagus cell line) and MDBK (bovine kidney epithelial cell line) at approximately 90% confluency were infected with 1 ml of tissue homogenate supernatant and incubated at 37 °C for one hour. The inoculum was removed and replaced with 5 mL OptiMEM, supplemented with Gentamicin (5 mg⁄mL)/Amphotericin B (125 µg⁄mL) and Penicillin (100 IU/mL)/Streptomycin (100 µg/mL). Cells were checked daily for the development of cytopathic effects. Virus titers were determined by performing TCID50 end-point dilution assays in triplicate using tenfold serial dilutions from (10^−1^ to 10^−11^) with TCID_50_/mL calculated using the Spearman and Kärber method [23].

### Detection of adenovirus mRNA

Suspected bovine adenovirus infection was confirmed using a pan-adenovirus degenerate PCR based detection method. Frozen spleen and liver tissue (200 mg) obtained at necropsy was homogenized in 500 µl PBS using ceramic beads in a FastPrep-24 5G homogenizer (MP Biomedical) and centrifuged at 12,000 RPM. 140 µl of tissue supernatant was used for automated RNA isolation using the QIAamp Viral RNA Mini Kit for the Qiacube (Qiagen) according to manufacturer’s instructions. Subsequently, a one-step RT-PCR kit (Qiagen) was used with primers of a consensus nested-PCR method for the detection of adenoviruses (Additional file 2) [24]. The amplified RT-PCR product of 321 bp was extracted using the Monarch® DNA Gel Extraction Kit (New England BioLabs), Sanger sequenced (Eurofins Genomics) and analyzed using BLAST (Basic Local Alignment Search Tool) with the GenBank NCBI nucleotide database.

### Next generation sequencing

A pool of liver and spleen tissue obtained from the calf with suspected adenovirus infection was processed for next-generation sequencing (NGS), as previously described [25]. A DNA library was prepared according to the manufacturer’s protocol (Nextera XT DNA Library Preparation Kit; Illumina) and sequenced on an Illumina NextSeq 550 platform with a NextSeq 500/550 High Output Kit v2 (2 × 75 bp paired end; Illumina). Bioinformatics analysis and retrieval of nearly complete consensus genome sequence was carried out using the CZ ID open-source pipeline designed for detection and monitoring of pathogens from raw FASTQ sequencing data [26, 27]. The genome termini sequences were completed using a modified rapid amplification of cDNA end (RACE) protocol (s) using BAdV7 specific primers (Additional file 2) in combination with 5’ adapters as described previously [28].

### Genome annotation and phylogenetic analysis

In silico prediction of gene sequences, analysis and visualization was performed using Geneious Prime® version 2021.2.2 (Biomatters, Ltd., Auckland, New Zealand). Genome annotations were predicted using the Genius Prime integrated annotation prediction tool and manually verified. The variable region between E4.1 and RH5 coding sequences was Sanger sequenced using BAdV7 specific primers (Additional file 2) and analyzed for repeats using the Geneious Prime integrated plugin “Repeat finder”. Pairwise alignments and visualization of BAdV-7 and OvAd-7 sequences was performed using the Genious Prime intergradet MAFFT pairwise aligner. For complete genome phylogenetic analysis, alignments of the adenoviral sequences was carried out using MAFFT multiple aligner [29] with maximum likelihood phylogenetic trees constructed using MEGA X (29). For complete genome phylogenetic analysis, a subset of twenty adenovirus complete genome sequences were selected. These included other members of the Atadenoviruses and representatives of all adenovirus genera. Phylogenetic trees were constructed using a GTR + G + I model using a bootstrap test of 500 bootstraps.

## Results

### Pathological, histological, and ultrastructural analysis

**Fig. 1:**
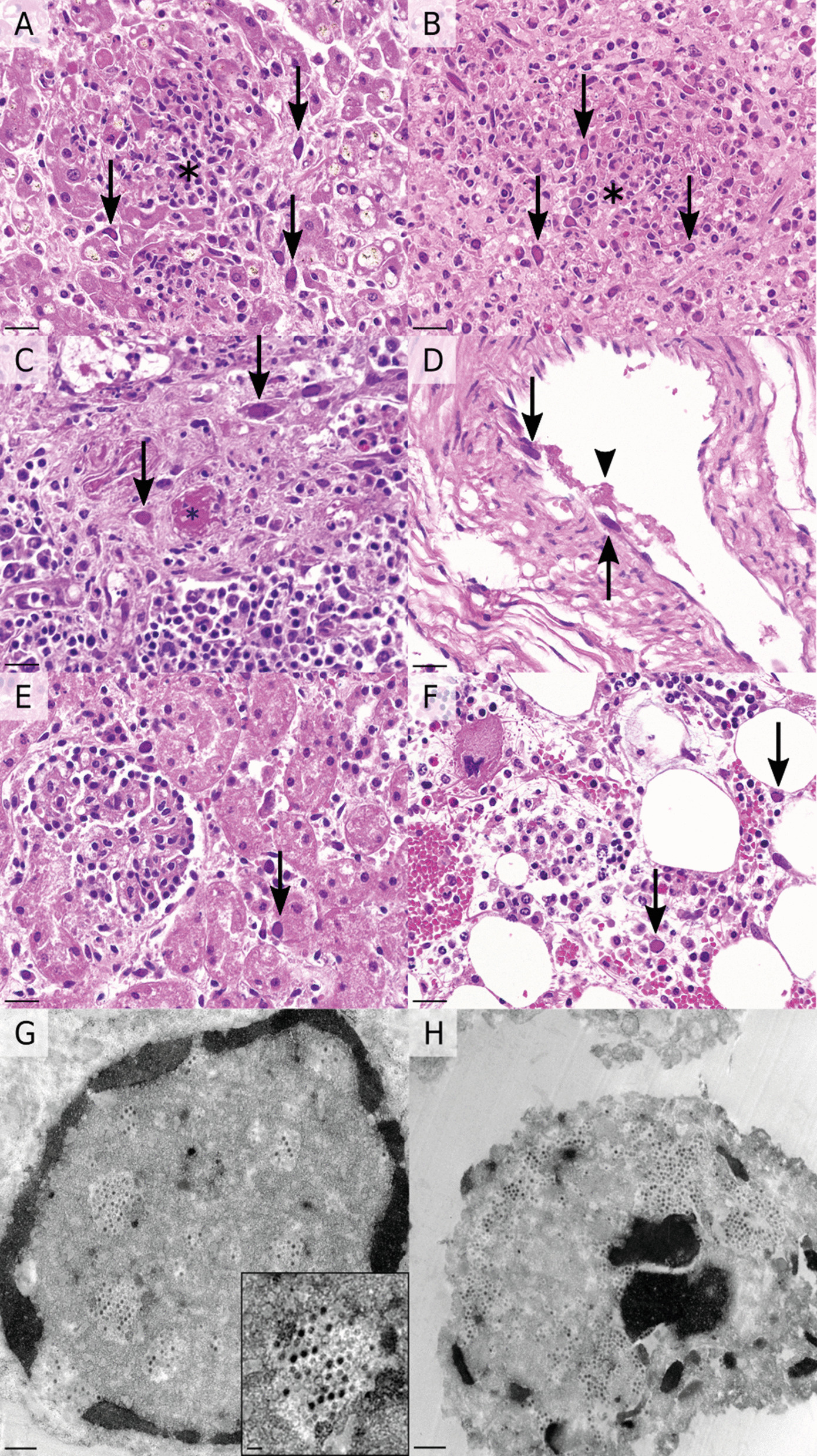
Histologic and ultrastructural findings in a calf with systemic adenoviral infection. **A**, **B** Necrotic foci (asterisks) within the liver **A** and spleen **B**, with numerous intralesional, amphophilic, intranuclear inclusions (arrows). **C** Lymph node with intravascular, fibrinocellular thrombi (asterisk), perifocal edema and associated intranuclear viral inclusions. All examined lymphoid tissues also showed generalized depletion. **D** Rete mirabile showing intrendothelial viral inclusions (arrows) and associated fibrin accumulation (arrowhead). **E**, **F** Viral inclusions not associated with overt histopathological lesions were detected in many other tissues, including kidney **E** and bone marrow **F**. **G** Electron microscopy of liver tissue showing nucleus with marginalized, condensed chromatin and intranuclear viral particles measuring ~ 70 nm in diameter and partly arranged in a crystalline array. **H** Similar viral particles are present within a necrotic cell in the spleen. **A**–**F**, **H** & **E** staining, scale bars: 20 µm. **G**, **H**, transmission electron microscopy, scale bars: 500 nm and 100 nm (insert)

Gross pathological examination of the calf at necropsy showed severe dehydration and catarrhal enteritis with watery contents in both the small and large intestines. In the outer umbilicus, a focal, suppurative inflammation was present, but the intraabdominal structures including umbilical vein, arteries and urachus, were unremarkable. Histopathologic examination of hematoxylin–eosin-stained sections revealed moderate to severe, necrotizing lesions in several tissues, most prominently in the liver and lymphoid organs. The liver showed randomly distributed foci of acute hepatocellular necrosis and multifocal thrombosis of small caliber blood vessels. At the periphery of the lesions, numerous cells with an endothelial morphology contained large, intranuclear, amphophilic inclusions, which distended the nuclei and peripheralized the chromatin, consistent with adenoviral inclusions. (Fig. 1A). In addition to the necrotizing lesions, a mild to moderate, multifocal, periportal lymphohistiocytic hepatitis was noted. Lymphoid organs, such as spleen, various lymph nodes, Peyer’s patches, tonsils, and thymus showed variable degrees of lymphoid depletion, multifocal necrotizing to necrosuppurative foci, and frequent fibrinous to fibrinocellular thrombi within small caliber blood vessels. These lesions were also often associated with intranuclear viral inclusions within endothelial cells (Fig. 1B, C). Identical inclusions were also found in endothelial cells of almost all other examined tissues, including kidney, bone marrow, adrenals, endocardium, *rete mirabile* and joint capsule. Occurrence of viral inclusions was occasionally accompanied by polymerized fibrin deposition or thrombosis, but in some organs, no associated morphologic lesions were present (Fig. 1D–F). Inclusions were also visible in the lamina propria of the intestines. To characterize the intranuclear inclusions, transmission electron microscopy was performed on paraffin-embedded liver and spleen tissue sections using the ‘pop-off technique’ [22]. In both organs, numerous intact and lytic nuclei contained obvious accumulations of viral particles (Fig. 1E, F). The virions had a diameter of approximately 70 nm and were frequently arranged in a crystalline array, consistent with the typical morphology of mature adenoviral particles. In addition to histopathology, further selected tests were performed using frozen tissue collected during necropsy (Additional file 1). Since the observed histologic lesions could also be caused by bacterial septicemia, spleen and liver samples were subjected to standard aerobic bacterial culture. In the liver, moderate amounts of *Listeria monocytogenes* and low amounts of Bacillus spp. were isolated. In the spleen, low amounts of Bacillus spp., coagulase-negative Staphylococci and E. coli bacteria were cultured.

### Identifcation, isolation and characterization of a BAdV-7 strain

The presence of intranuclear inclusions in endothelial cells in multiple tissues was suggestive of a systemic adenovirus infection. This was confirmed by performing a degenerate pan-adenovirus RT-PCR to detect adenovirus mRNA in spleen and liver tissue homogenate. Sanger sequencing of a positive RT-PCR product showed nucleotide similarity of 75% to the hexon gene of ovine adenovirus 7 (GenBank accession no. U40839.3). The specific identity of this adenovirus sequence was ascertained by performing NGS on a pooled library of spleen and liver tissue. The resulting NGS reads were uploaded to CZ ID, a cloud-based, open-source bioinformatics platform used for identification of microbial pathogens. A total contig of 29,756 bp was recovered by de novo assembly with BLASTed with NCBI nucleotide and protein databases, aligning the contig with previously published Bovine Atadenovirus 7 sequences (GenBank accession nos. AF238232, X53989, U57335, AY288821, AY288815). A de novo assembly of NGS reads and Sanger sequencing of genome termini ends by use of RACE protocols revealed a total genome length of 29,973 bp, with an average G + C content of 33.6% (BAdV-7, S427/8, GenBank accession No. OM677816). Characteristic adenoviral inverted terminal repeat (ITR) at the extreme genome ends were revealed to be 36 bp in length. In silico annotation prediction reveals the presence of thirty coding sequences (Table 1).

**Table 1:** Genome annotiation of BadV-7 strain S427/18

Region	Annotation	Minimum	Maximum	Length	Intervals	Direction
ITR repeat region	ITR	1	36	36	1	Forward
p32K CDS	CDS	235	1155	921	1	Reverse
LH1 CDS	CDS	1197	1550	354	1	Forward
LH2 CDS	CDS	1535	1909	375	1	Forward
E1B 55 K CDS	CDS	1959	3107	1149	1	Forward
IVa2 CDS	CDS	3115	4209	1095	1	Reverse
pol CDS	CDS	4194	7430	3237	1	Reverse
pTP CDS	CDS	7412	11,924	1788	2	Reverse
52 K CDS	CDS	9203	10,198	996	1	Forward
pIIIa CDS	CDS	10,183	11,895	1713	1	Forward
III protein CDS	CDS	11,937	13,295	1359	1	Forward
pVII CDS	CDS	13,337	13,672	336	1	Forward
pX CDS	CDS	13,694	13,906	213	1	Forward
pVI CDS	CDS	13,947	14,582	636	1	Forward
hexon CDS	CDS	14,545	17,331	2787	1	Forward
protease CDS	CDS	17,328	17,936	609	1	Forward
DBP CDS	CDS	17,940	19,085	1146	1	Reverse
100 K CDS	CDS	19,105	20,991	1887	1	Forward
22 K CDS	CDS	20,858	21,055	198	1	Forward
33 K CDS	CDS	20,858	21,367	408	2	Forward
pVIII CDS	CDS	21,399	22,052	654	1	Forward
U Exon	CDS	22,064	22,228	165	1	Reverse
fiber CDS	CDS	22,236	23,642	1407	1	Forward
E4.3 CDS	CDS	23,645	24,298	654	1	Reverse
E4.2 CDS	CDS	24,298	24,957	660	1	Reverse
E4.1 CDS	CDS	24,957	25,388	432	1	Reverse
RH5 CDS	CDS	26,990	27,619	630	1	Reverse
RH4 CDS	CDS	27,622	28,059	438	1	Reverse
RH3 CDS	CDS	28,280	28,762	483	1	Reverse
RH2 CDS	CDS	28,789	29,163	375	1	Reverse
RH1 CDS	CDS	29,269	29,865	597	1	Reverse
ITR repeat region	ITR	29,938	29,973	36	1	Forward

**Fig. 2:**
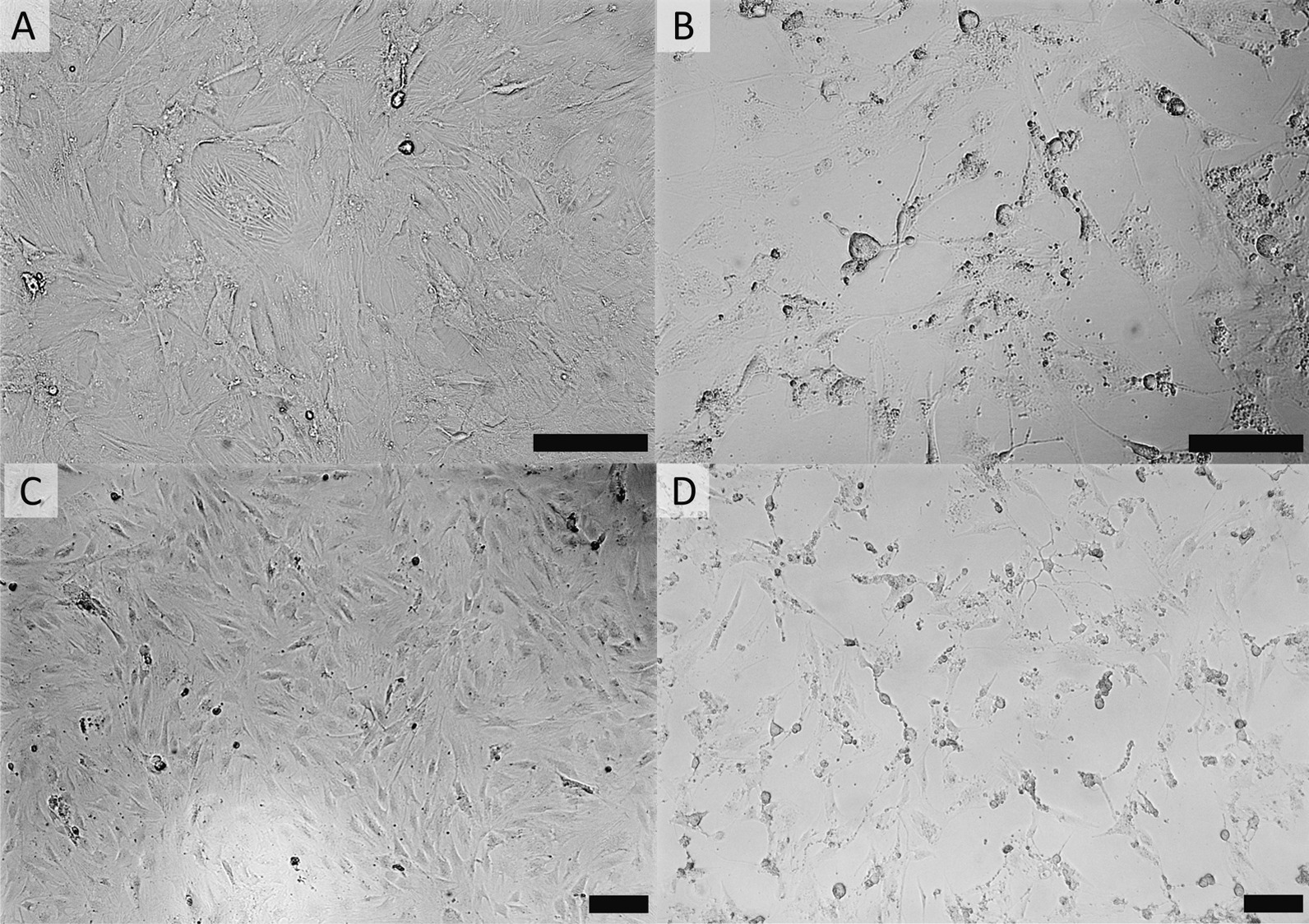
Cytopathic effects (CPE) induced by BAdV-7 infection of bovine esophagus cells (KOP-R). **A**–**C** No CPE is observed in mock infected KOP-R cells at low **A** or high **C** magnification whereas BAdV-7-infected KOP-R cells show extensive CPE at low **B** or high **D** magnification including cell swelling and clumping. Photomicrographs were taken on a Leica DMi8 microscope. Scale bars, 150 µm

Given the paucity of avaliable BAdV-7 strains for use in in vitro and in vivo studies, we attempted to isolate virus by overlay of homogenized and filtered liver tissue onto KOP-R and MDBK cells. Cytopathic effects (CPE) were not observed in infected MDBK cell monolayers (data not shown) or in mock-infected KOP-R cells (Fig. 2A–C). Consistent CPE consisting of cell-swelling, -clumping, and -rounding could be observed in KOP R cell monolayers three days post-infection with tissue homogenate (Fig. 2B–D). Titration of the BAdV-7 virus stock produced on KOP-R cells by 50% Tissue Culture Infectious Dose assay (TCID50) showed that the titer of the first passage virus stock was 5.62 × 10^6^ TCID_50_/ml.

**Fig. 3:**
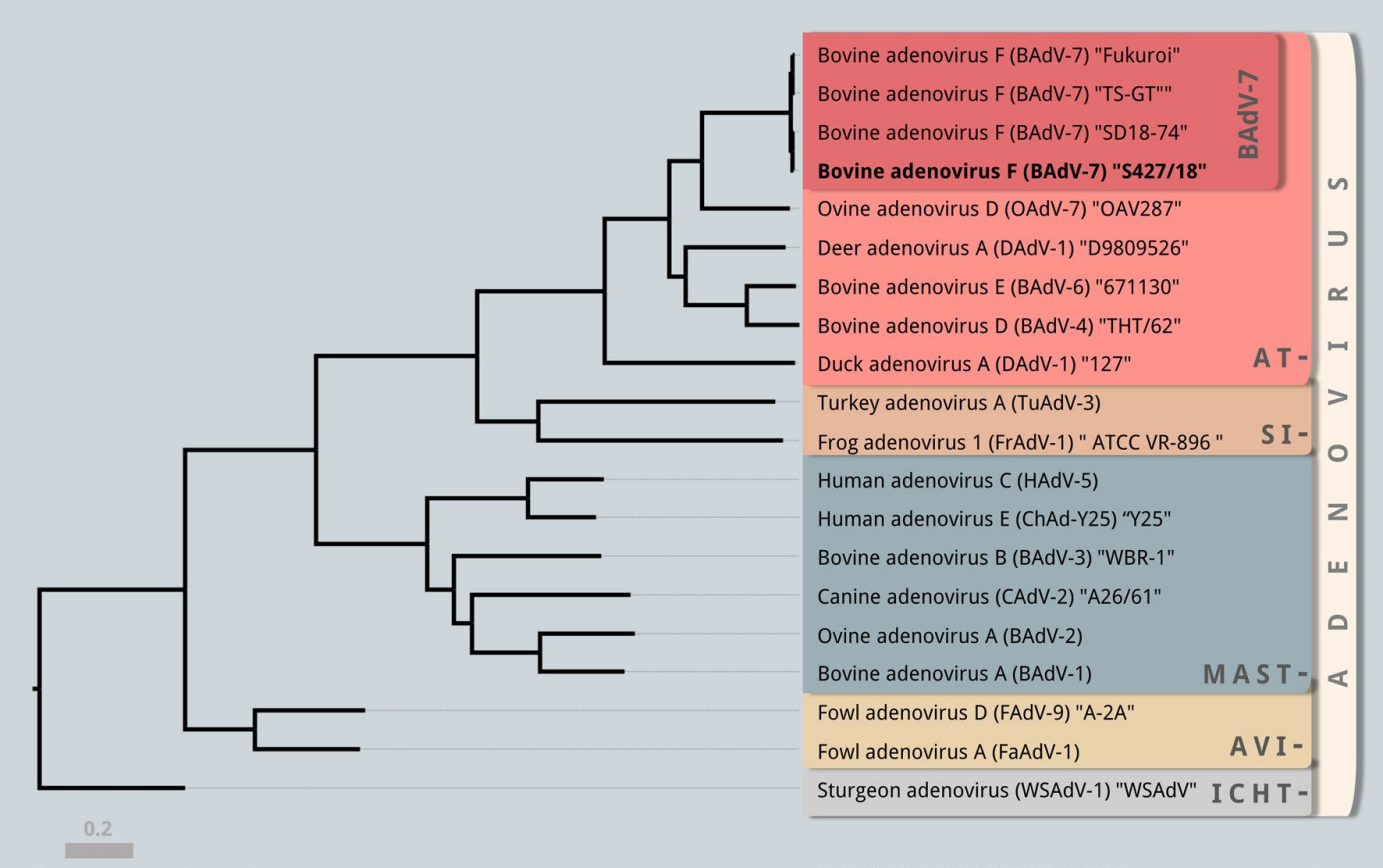
Phylogenetic Analysis of complete Adenovirus genomes. Maximum likelihood phylogenetic analysis was performed using a subset of complete genome sequences representing all Adenovirus genera (Atadenovirus, Siadenorius, Mastadenovirus, Aviadenovirus, Ichtadenovirus). Tip labels represent the official species nomenclature, along with abbreviation of the virus in brackets, and the respective strain name. The newly identified BAdV-7 strain “S427/18” (boldface (bold type) is placed within the genus of Atadenovirus (red). “Strain S427/18” shares closest homology to the recent US strain “SD18-74”. Complete genome sequence phylogeny confirms that all sequences of BAdV-7 form their own distinct clade within the genus Atadenovrius. Phylogenetic analysis was performed using MEGA X (29) and a GTR + G + I model with 500 bootstraps. The grey scalebar indicates the number of substitutions per sites with branch lengths being proportional to genetic distances. Bootstrap values of the major nodes are depicted in grey

**Fig. 4:**
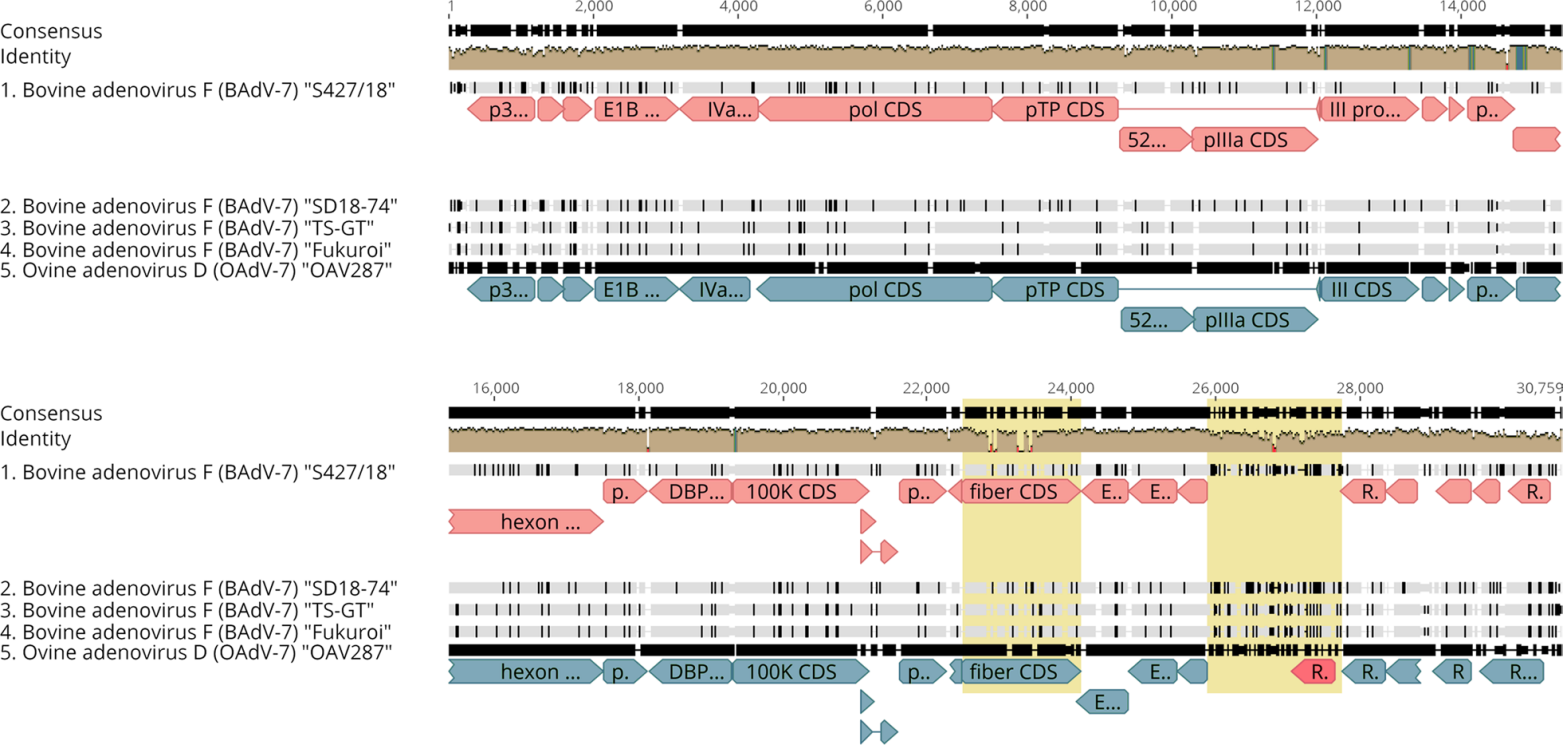
Annotation and alignment of bovine adenovirus type 7 (BAdV-7) and ovine adenovirus type 7 (OAdV-7) genomes. An alignment is shown for the BAdV-7 strains Fukuroi, TS-GT, SD18-74, and S427/18 (GenBank accession nos. LC597488, LC606503, MN901942, OM677816) and the Ovine adenovirus D (OAdV-7) prototype strain OAV287 (GenBank accession no. OAU40839). Arrows represent the annotated open reading frames of respective species, with BAdV-7 in pink and OAd7 in blue. Brown histogram represents sequence identities among selected sequences. The yellow boxes highlight the most variable regions of the alignment, the fiber gene shows highest level of interspecies sequence variability. The interval position 25,389–27,017 nt between E4.1 CDS and RH5 CDS is the genome region displaying the highest level of intraspecies sequence variability. The red arrow located in the OAdV-7 genome annotation highlights the open reading frame RH6 that is missing in all BAdV-7 genome sequences

Maximum likelihood phylogenetic analysis of complete adenoviral sequences places the newly identified strain within the genus of Atadenoviruses (Fig. 3). BAdV-7 strain S427/18 shows closest nucleotide identity of 99.16% to a recent USA isolate with 98.60 and 98.52% sequence identity observed with the Japanese prototype Fukuroi strain and the TS-GT vaccine strain respectively (Table 2). Our analyses confirms that BAdV-7 shares closest sequence homology to ovine adenovirus 7 (OAdV-7), with the newly identified S427/18 strain sharing 64.64% sequence identiy with the ovine Adenovirus 7 prototype OAV287 strain. The most variable regions of the BAdV-7 genomes were identified as the sequence encoding the fiber protein and the non-coding region bewteen E4.1 and RH6 (Fig. 4). All fiber genes from BadV-7 strains have a length of 1407 bp (469 aa) whereas OAdV-7 has a fiber length of 1632 bp (544 aa). The fiber gene represents the region of highest interspecies variability. The S427/18 strain shares only 39.56% aa identity with OAdV-7, whereas BadV-7 intraspecies variability ranges from 99.57 to 99.79% aa identity.

**Table 2:** Nucelotide sequence identity (%) matrix of full genome sequences of bovine adenovirus type 7 and ovine adenovirus type 7

	BAdV-7 S427/18	BAdV-7 SD18-74	BAdV-7 TS-GT	BAdV-7 Fukuroi	OAdV-7 OAV287
BAdV-7 S427/18		99.16	98.52	98.60	64.68
BAdV-7 SD18-74	99.16		98.99	99.07	64.65
BAdV-7 TS-GT	98.52	98.99		99.92	64.69
BAdV-7 Fukuroi	98.60	99.07	99.92		64.74
OAdV-7 OAV287	64.68	64.65	64.69	64.74

**Fig. 5:**
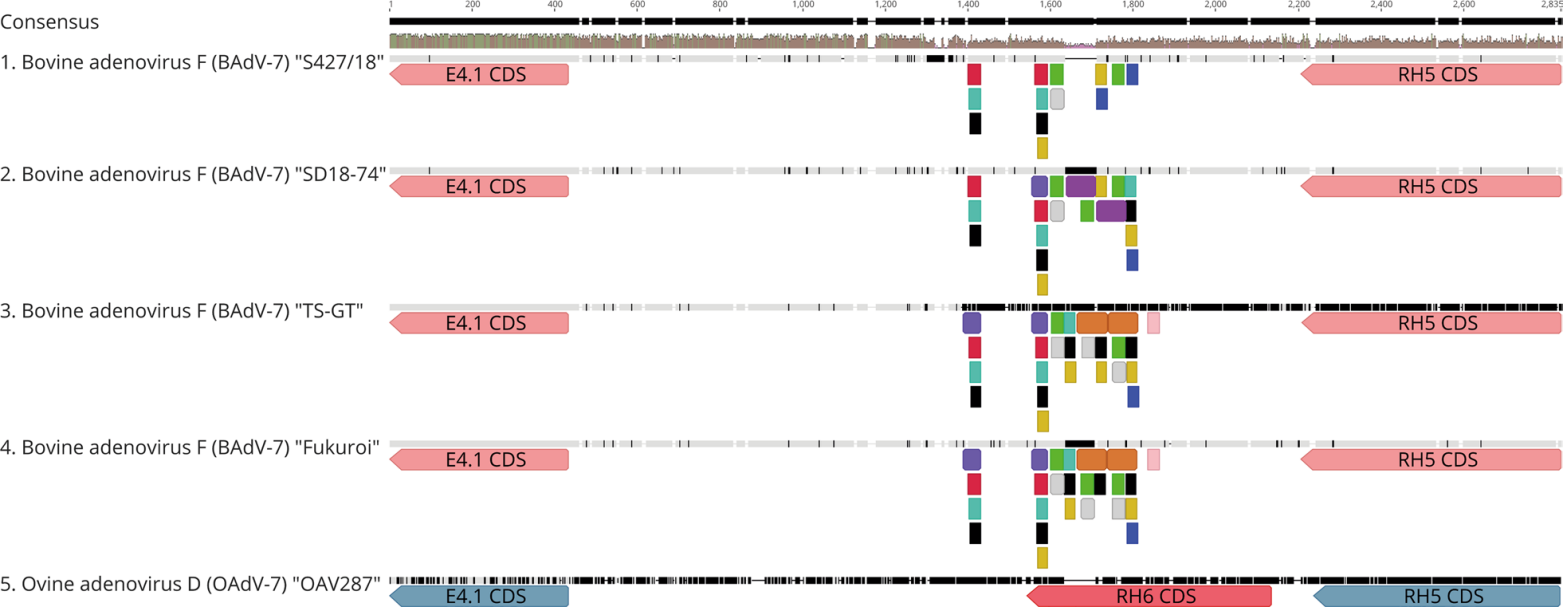
Overview of the non-coding tandem repeat region present between E4.1 CDS and RH5 CDS. An alignment is shown of the non-coding region between positions 25,389 and 27,017 bp of the BadV-7 S427/18 strain genome in comparison to the equivalent region of the Ovine Adenovirus 7 (OAdV-7) genome. The arrows represent the E4.1 and RH5 open reading frames of of BAdV-7 (pink) and OAdV-7 (blue). The boxes represent the tandem sequence repeats of varying length. Each color represents a unique sequence. The older Fukuroi strain and the TS-GT vaccine strain (based on Fukuroi) are identical in this region and share the same repeat sequences. The newer strains are variable to each other and to the original Japanese strains. The newly identified German strain S427/18 has an insertion of 17 bp in comparison to the three published BAdV-7 strains and a deletion of 76 bp

Alhough BAdV shares closest homology to OAdV-7, we confirmed that the RH6 gene which is present in the OAdV-7 genome, is absent in the genomes of BAdV-7 (Fig. 5). Interestingly, this region between E4.1 and RH5 coding sequences has the highest levels of intraspecies sequence variability within BAdV-7 genome sequences, with multiple start and stop codons, but no predicted open reading frame. Instead, multiple internal tandem repeats are present which vary in length from 25 to 71 nucleotides. The Japanese prototype Fukuroi strains and the TS-GT vaccine strain share 100% sequence identity in this region, whereas the newly identified S427/18 strain and the recent SD18-74 strain from the USA share 91.03% identity. The S427/18 strain also has an insertion of 17 bp when compared to the other three published BAdV-7 strains and a deletion of 76 bp which is unique to this BadV-7 strain.

## Discussion

Bovine Adenoviruses are well known pathogens of cattle, but BAdV-7 remains relatively uncharacterized with respect to molecular epidemiology and pathogenesis. In this study, we report the first molecular characterization of a European BAdV-7 strain, along with histopathological and ultrastructural analysis performed on tissues from a deceased a calf in northern Germany. Bovine adenoviruses have a worldwide distribution and are frequently detected in healthy animals, but also in animals with severe enteric and respiratory disease [5, 6, 30]. Pathomorphology is characterized by necrotizing lesions within the lower gastrointestinal tract with associated lymphoid depletion and necrosis in Peyer’s patches and regional lymph nodes [8, 10, 11, 31]. Viral inclusions have been demonstrated within swollen endothelial cells in association with necrotic foci and rarely in enterocytes. Infection of endothelium with consecutive vascular injury and thrombosis is presumably a prerequisite for severe disease and the primary cause of necrotizing lesions. BAdV-10 has been implicated as a cause of fatal enteritis in cattle from Northern Ireland and New Zealand and a strain-specific digoxigenin-labelled DNA probes has been used to detect in intestinal endothelial cells using in situ hybridization [8, 32].

In the current case, autolytic changes precluded a definite assessment of histologic lesions within the gastrointestinal tract, but viral inclusions were observed in cells with an endothelial morphology in the lamina propria of the intestine. Moreover, necrotizing lesions and inclusions were particularly severe/abundant in mesenteric lymph nodes. Therefore, the lower gastrointestinal tract seems a plausible primary infection site in the animal. In addition to the intestine, adenoviral inclusions were observed in many other organs and were associated with necrosis in the liver, spleen, and other lymphoid organs throughout the body suggesting a systemic disseminated infection. Viral inclusions have been demonstrated in extra-intestinal sites in animals with adenoviral enteritis, but usually not in association with major necrotizing lesions [8, 10, 32, 33]. Generalized vasculitis associated with BAdV infection has been described in neonatal calves with a systemic disease termed weak calf syndrome (WCS) [13, 14, 33, 34]. A causative relationship between WCS and BAdV was demonstrated by experimental infections with a BAdV-5 strain isolated from an infected calf, which reproduced the disease phenotype [14]. In addition, experimental intra-amniotic infection with a BAdV-7 strain resulted in two prematurely born calves with a systemic disease distinct from WCS and one stillbirth [35]. Therefore, intrauterine infection with bovine adenoviruses should be considered as an etiologic differential for neonatal disease and mortality in calves.

The presence of necrotizing lesions in liver and lymphoid organs may have occurred because of systemic pathology induced by co-infecting pathogen causing bacterial septicemia. Several bacterial species were cultured from spleen and liver tissue of this animal but apart from *Listeria monocytogenes*, the other bacterial isolates were considered likely to be contaminants and not related to the observed pathology. *L. monocytogenes* can cause septicemia in neonatal calves, which is characterized by multisystemic necrotic lesions and micro abscesses and usually results from intrauterine infection [36, 37]. Therefore, it remains uncertain whether adenoviral infection was the primary insult in this case or whether a primary listeria infection predisposed the calf to systemic dissemination of BAdV-7. In some tissues, viral inclusions were demonstrated in endothelial cells without associated obvious pathological alterations. Therefore, systemic BAdV-7 infection might also represent an epiphenomenon without clinical significance in the animal. To test the significance of a Listeriosis, we have performed Listeria specific immune histochemistry on several tissues. Though Listeria was indeed present in mandibular lymph nodes, this pathogen could not be found in the spleen or in any other tissue.

The pathological analyses and pan-adenovirus degenerate RT-PCR strongly indicated that a bovine adenovirus may be present within the tissues of the calf under investigation. This was confirmed to be BAdV-7 following analysis of NGS reads obtained from a DNA library prepared from liver and spleen tissue samples. The recovery of the first full-length genome sequence of a European BAdV-7 strain (S427/18) directly from clinical material means that this is a true wild-type virus sequence without the possibility of mutations due to propagation in primary or transformed cells. Isolation of BAdV-7 has previously been restricted to primary bovine testicular cells, and bovine and ovine primary nasal turbinate cells [4, 15]. We have extended this list of susceptible cells by isolating BAdV-7 in KOP-R cells, a finite bovine eosophagus cell line with readily detected CPE on day three-post-infection. This tractable strategy may have increased utility for future in vitro and in vivo studies than the derivation of fresh primary cell cultures for use in isolating and titrating BAdV-7 strains from clinical material.

Phylogenetic analysis of the newly derived full-length sequence of BAdV-7 strain S427/18 agrees with previous phylogenetic analyses of sequences derived from the hexon, DNA polymerase and penton base genes [3, 4, 20]. These studies have reported that the BadV-7 and OAdV-7 predicted aa sequences of the DNA polymerase, penton base and hexon genes share sequence identity of 67, 84, and 86% respectively. Our data also supports a recent analysis highlighting interspecies variability within the fiber gene (39.56% aa sequence identity to OAdV-7) [4]. We also identified the BAdV-7 genome region displaying the highest levels of intraspecies nucleotide sequence variability of BadV-7 to be in the genome region between the genes E4.1 and RH5. As previously described, this region is missing the gene RH6, which is present in the OADV-7 genome [4, 38], but instead contains various internal tandem repeats of varying length (25–71 bp). It is important to note, that this repeat region of BAdV-7 could be a result of de novo or reference assembly errors. To exclude this possibility, we Sanger sequenced this genome region to confirm the authenticity of the tandem repeats. Intra-species variability is only observed upon comparison of older Japanese BAdV-7 strains to current US and European strains, suggesting that although this region is non-coding, it may have a role in the evolution of the virus. The function(s) of this non-coding region are unknown and require further investigation [4, 39]. Such repeat sequences in non-coding regions are often observed in other dsDNA viruses [40]. For instance, the inverted terminal Repeat (ITR), is present in all adenoviruses [38], but within the genus Atadenovirus, this tandem repeat region appears to be unique to BAdV-7 genome sequences. Tandem repeat regions have previously been described in other adenoviruses of other genera, including fowl adenovirus type 9, canine adenovirus type 1 vaccine strain CLL and mouse adenovirus type 1 [41–43]. Interestingly, the site between the E4 and RH transcription units of OAdV-7 vector system has been shown to be a suitable site (site III) for insertion of foreign DNA and is stable over several passages of the recombinant virus in cell culture. Furthermore, the deletion of approximately 2 kb of the OAdV-7 genome encompassing this region did not significantly interfere with virus growth [44, 45]. The role of this variable region in the BAdV-7 genome with respect to virus evolution, and necessity or dispensability for virus replication in vivo needs further clarification. We further advocate for active molecular epidemiological surveillance of BAdV-7 to determine its true prevalence and clinical significance for cattle farms. Additional in vitro and in vivo characterization is also required to clarify the cellular tropism and disease course of BAdV-7 and further explore the role of co-infecting bacterial and/or viral pathogens in exacerbating levels of morbidity and mortality in infected cattle.

## Supplementary Information

**Additional file 1.** Diagnostic tests performed on frozen tissue collected during necropsy of a calf.

**Additional file 2.** Primers sequences used in this study.

## Data Availability

The full-genome sequence of the bovine adenovirus type 7 strain characterized in this study was submitted to GenBank under accession number OM677816. All other data generated or analyzed during this study are included in this published article and the supplementary information files.
