# Supplementary material for: Molecular characterization of a bovine adenovirus type 7 (Bovine Atadenovirus F) strain isolated from a systemically infected calf in Germany
Source: Virol J. 2022 May 24;19:89. doi: 10.1186/s12985-022-01817-y (PMC9131638; doi:10.1186/s12985-022-01817-y)
Supplement: Supplementary file 1 — Additional file 1. Diagnostic tests performed on frozen tissue collected during necropsy of a calf. [file 12985_2022_1817_MOESM1_ESM.docx]

**Additional file 1.** Diagnostic tests performed on frozen tissue collected during necropsy of a calf.

| Pathogen | Tissue | Method | Result |
| --- | --- | --- | --- |
| Viruses |  |  |  |
| Adenoviruses | Liver, spleen | PCR | Positive |
| Herpesviruses | Liver, spleen | PCR | Negative |
| BTV* | Spleen | PCR | Negative |
| BHV-1* | Lung, lymph node | Immunofluorescence, cell culture | Negative |
| BVDV* | Spleen, tonsil, kidney, lymph nodes, parotid gland | Immunofluorescence, cell culture | Negative |
| Rota-, Coronaviruses** | Small intestine | ELISA | negative |
| Bacteria |  |  |  |
| Aerobes | Liver | Culture | L. monocytogenes (++); Bacillus spp (+) |
|  | Spleen | Culture | Bacillus spp., Coagulase-negative staphylococci, E.coli (+) |
| Salmonella* | Small intestine | Culture | negative |
| E. coli K99** | Small intestine | ELISA | negative |

* performed at the Lower Saxony State Office for Consumer Protection and Food Safety

** performed at the Institute of Virology, Veterinary University Hannover
