# Supplementary material for: Molecular characterization of a bovine adenovirus type 7 (Bovine Atadenovirus F) strain isolated from a systemically infected calf in Germany
Source: Virol J. 2022 May 24;19:89. doi: 10.1186/s12985-022-01817-y (PMC9131638; doi:10.1186/s12985-022-01817-y)
Supplement: Supplementary file 2 — Additional file 2. Primers sequences used in this study. [file 12985_2022_1817_MOESM2_ESM.docx]

**Additional file 2.** Primers sequences used in this study.

| **Name** | **Sequence (5'-3')** | **Direction** | **Reference** |
| --- | --- | --- | --- |
| Ad_polFouter | TNMGNGGNGGNMGNTGYTAYCC | forward | Pan-adenovirus PCR primers: Wellehan *et al*. (2004) (24) |
| Ad_polRouter | GTDGCRAANSHNCCRTABARNGMRTT | reverse |  |
| Ad_polFinner | GTNTWYGAYATHTGYGGHATGTAYGC | forward |  |
| Ad_polRinner | CCANCCBCDRTTRTGNARNGTRA | reverse |  |
| RACE_BAdV7_L95- | GCACTTCCTGCAGTTCCATTAGC | reverse | RACE primers for adenovirus ITRs:  this study |
| RACE_BAdV7_L163- | GAGGATTTATGAGAAACCTTGCAGC | reverse |  |
| RACE_BAdV7_T29545+ | GCCACATCTACGTTGTCTAAGTATTGC | forward |  |
| RACE_BAdV7_T29462+ | GACTGAATCGTTTCCAATACTGC | forward |  |
| BAdV-7_25336+ | AAGGGCATATAATTCAGAGC | forward | Sequencing of region between RH5 and E4 CDS:  this study |
| BAdV-7_27130- | TTAATCATGCGCAGTATACTGC | reverse |  |
| BAdV-7_27177- | TTGTCGTCTCTTCTTACTG | reverse |  |
| BAdV-7_26525- | GGGTCTAATTATAACTGCTGTAAATAA | reverse |  |
| BAdV-7_26499+ | TTATTTACAGCAGTTATAATTAGACCC | forward |  |
